# Influence of Molecular Conformations and Microstructure on the Optoelectronic Properties of Conjugated Polymers

**DOI:** 10.3390/ma7032273

**Published:** 2014-03-19

**Authors:** Ioan Botiz, Natalie Stingelin

**Affiliations:** 1 Nanobiophotonics and Laser Microspectroscopy Center, Faculty of Physics and Interdisciplinary Research Institute in Bio-Nano-Sciences, Babes-Bolyai University, Treboniu Laurian Str. 42, Cluj Napoca 400271, Romania; 2 Department of Materials, Imperial College London, Exhibition Road, London SW7 2AZ, UK; E-Mail: natalie.stingelin@imperial.ac.uk

**Keywords:** conjugated polymer materials, nanostructuring, molecular conformation, morphology, microstructure, optoelectronic properties, organic devices

## Abstract

It is increasingly obvious that the molecular conformations and the long-range arrangement that conjugated polymers can adopt under various experimental conditions in bulk, solutions or thin films, significantly impact their resulting optoelectronic properties. As a consequence, the functionalities and efficiencies of resulting organic devices, such as field-effect transistors, light-emitting diodes, or photovoltaic cells, also dramatically change due to the close structure/property relationship. A range of structure/optoelectronic properties relationships have been investigated over the last few years using various experimental and theoretical methods, and, further, interesting correlations are continuously revealed by the scientific community. In this review, we discuss the latest findings related to the structure/optoelectronic properties interrelationships that exist in organic devices fabricated with conjugated polymers in terms of charge mobility, absorption, photoluminescence, as well as photovoltaic properties.

## 1. Introduction

Conjugated polymers are interesting materials due to both their semiconducting properties and highly tunable molecular ordering that can proceed in a hierarchical way on multiple lengthscales ranging from nanometers to macroscopic dimensions, leading to a vast variety of nano-, meso-, and micro-structures [1–6]. This breath of architectures that can be realized can be designed to target a plethora of applications, amongst others, in the fields of nanolithography [7], controlled drug delivery [8], organic photovoltaics (OPVs) [9–13], organic light-emitting diodes (OLEDs) [14–18], and organic field-effect transistors (OFETs) [1–4,19–22].

Thin, active films of conjugated polymers are associated with a multitude of fundamental physical phenomena including exciton diffusion [23] and separation [24], charge transport [25,26] and carrier migration/mobility [27], exciton and charge recombination [28], as well as other interactions of excitons with the polymer chain/network, their trapping and loss [27], *etc.* These internal phenomena are all strongly dependent on the molecular conformations and longer-range arrangement adopted by the conjugated polymer molecules at all lengthscales [25,28,29]. The relation between this molecular arrangement and structural features adopted by the conjugated polymer chains, and their resulting optoelectronic properties, were investigated extensively in the last few years and are currently being explored by many research groups [25,26,28–35] in order to further understand—and eventually improve—the performance of organic devices based on conjugated polymers.

Here, we are going to review recent scientific advances that are emphasizing some of the structure/processing/optoelectronic properties interrelationships that are of key importance for the driving of organic devices based on conjugated polymers, including their charge-transport properties (with focus on charge transport measured in OFETs), absorption, and photoluminescence (PL) characteristics as, e.g., exploited in active layers of devices such as OLEDs and sensors, as well as the photovoltaic processes relevant in organic solar cells.

## 2. Influence of Molecular Conformation, Order and Chain Interconnectivity on the Charge Transport Properties of Polymer Field-Effect Transistors

Charge transport is a fundamental phenomenon that governs the functionality of all organic devices including OFETs. Therefore, understanding this phenomenon has a significant importance not only from a scientific but also from a technological and applicative point of view. The lack of continuous pathways throughout a conjugated active layer, e.g., due to the presence of structural defects in the organic material (e.g., caused through chain folding or disorder in the material network), disturbs the transport of charge carriers. Accordingly, free charges can get trapped at structural defects and recombine before being transported to the electrodes, leading to poor transport. Instead, a perfect pathway with no structural defects could act as a “fast-transport lane” with no energy dissipation and, possibly, with fewer charge recombination events.

In general, for polymer chains with defects, a weak disorder can increase the interaction of the charge carrier with the environment over longer distances leading to non-coherent transport [36]. Coherent charge transport dominates up to a certain length, above which conformational disorder breaks the coherence and activates hopping transport. Therefore, transport of charge carriers can take place both along single chains (intra-chain transport) and/or via an inter-chain process. The intrinsic structural anisotropy, hence, can be expected to lead to an anisotropic character of charge transport in active layers of organic device fabricated with conjugated macromolecular species.

**Figure 1.**
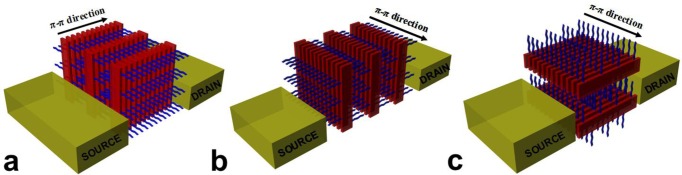
Schematic representation of model architectures comprised of a conjugated polymer that would be ideally suited to measure in an OFET geometry the charge-carrier mobility along the: (**a**) π-π inter-chain direction; (**b**) side-chain direction and (**c**) intra-chain direction.

In order to clearly establish whether intra-chain or inter-chain transport dominates in a given architecture, *idealized model systems of controlled molecular conformations* and *order* are needed (Figure 1). In such structures, all conjugated molecules ideally would adopt a unique conformation/orientation at all lengthscales, with all chains being fully extended and planarized without torsional defects. However, measuring charge transport in such model systems is highly challenging. An OFET is a type of transistor that is used to control the mobility of a specific charge carrier (electron or hole) throughout a channel in a material made of organic semiconductor material. Field-effect (carrier) mobility μ_FET_ describes how fast an electron or a hole is travelling through a semiconductor material and can be inferred from the OFET geometry via two types of measurements. One measurement is based on saturation-mode and consists in increasing the drain-source voltage until the current saturates for each fixed gate voltage. A second, measurement is based on the so-called ohmic-mode when the transistor is operated in the linear regime. Below, we will discuss the most recent attempts to correlate certain structural features with observed charge transport phenomena in terms of OFET mobility with focus on architectures where the conjugated polymers adopt specific molecular conformations and packing.

One of the most widely studied conjugated polymers is poly(3-hexylthiophene) (P3HT). When cast or coated on a substrate and further used in an OFET geometry, this polymer shows a typical μ_FET_ hole mobility of ~10^−3^ up to 10^−1^ cm^2^V^−1^s^−1^ [37–41]. Using processing methods that rely on the nature and/or quality of solvent [42], allow exploitation of controlled crystallization phenomena, e.g., via high-pressure crystallization or directed crystallization [31,39,43], or permit utilization of post-deposition procedures, such as annealing at elevated temperatures [44], or in controlled solvent vapor atmosphere [45–48], one can readily vary the chain conformations and arrangements of a given polymer, leading to a multitude of morphologies and microstructures with various structural properties. As a consequence, a large variety of optoelectronic properties and a broad range of charge-carrier mobilities can be obtained for many macromolecular semiconductors, including P3HT. For example, Newbloom *et al.* [49] recently obtained a mesoscale morphology of P3HT fibers crystallized through colloidal self-assembly in various aromatic solvents. Although the measured mobility was still rather poor compared to other reports, this study suggested that control of the degree of crystallinity and the resulting network structure of P3HT crystallized in solution before spin casting could be used to increase the charge carrier mobility in devices. This view is supported by Boudouris *et al.* [43] Using a poly(3-(2’-ethyl)hexylthiophene) (P3EHT) system with a lower melting temperature and a liquid crystal transition, these authors have demonstrated that OFET hole mobilities significantly increased (~60-fold with respect to the amorphous film) with the polymer’s degree of crystallinity due to the formation of a thin-film crystalline network in an amorphous matrix. The best value obtained for μ_FET_ was, however, still rather poor, in the range of 10^−4^ cm^2^V^−1^s^−1^.

Another factor that is critical to consider when correlating charge transport with structure is the anisotropic nature of organic semiconductors whereby one has to distinguish between intra-chain transport *vs.* hopping along the π-π stack (as alluded to above). In order to assess the anisotropic nature of the charge transport in terms of mobility, one can, for instance, prepare top-contact OFETs with the channel positioned at different angles along the aligned structures, e.g., with nanostructured objects (for example, fibers) parallel to the channel length in the so called parallel devices or with such objects perpendicular to the channel length in the so called perpendicular devices. In 2010, Xiao *et al.* [48] reported single crystals of P3HT that were comprised of polymer chains with their main axis parallel to the substrate, obtained via vapor annealing and controlling the solvent evaporation rate. While possessing rather low inter-chain mobility in the π-π stacking direction (μ_FET_ ~ 1.57 × 10^−3^ cm^2^V^−1^s^−1^), these crystals showed highly anisotropic electrical properties. Such a high in-plane anisotropic behavior was previously observed by Jimison *et al.* [39] in directionally crystallized thin films. It was indicated that the anisotropy was a result of grain boundaries between and along the P3HT crystalline fibers. In a later report, using time-of-flight measurements, Müller *et al.* [31] have shown that crystals of P3HT obtained under high pressure conditions have a slightly improved mobility which they attributed to the increased lamellar thickness of the crystalline moieties. Note here that time-of-flight mobility μ_TOF_ is obtained using a method that is different from OFET measurements and is based on estimation of the time taken by the carriers (electrons or holes) photo-generated close to one electrode to travel across the semiconducting material and reach the other collecting electrode.

**Figure 2.**
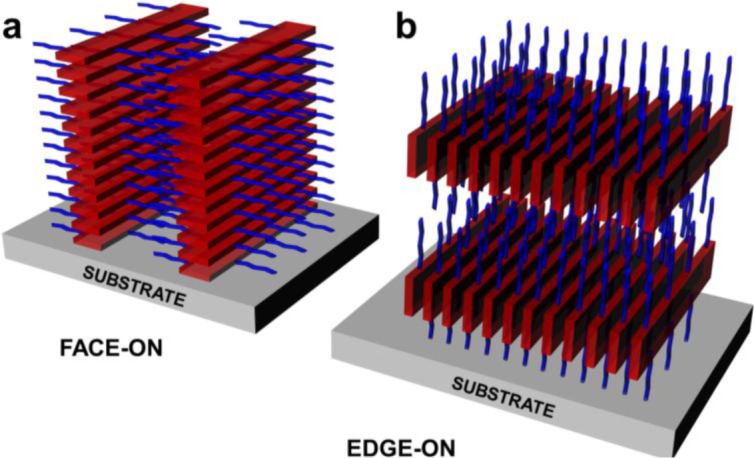
Classical representation of (**a**) face-on and (**b**) edge-on orientations with red rectangular shapes denoting the conjugated backbone and blue arms symbolizing the polymer side chains (in a non-interdigitated conformation).

An efficient and original approach to control polymer chain orientation with respect to the substrate on large-scale was recently developed and consists in combining mechanical rubbing with directional epitaxial crystallization [50]. For example, after rubbing, polymer chains of P3HT [50] or conjugated poly(2,5-bis(3-dodecyl-2-yl)thieno[3,2-*b*]thiophene) [51] align parallel to the rubbing direction and subsequently, crystalline domains undergo orientation changes from edge-on to face-on orientation (Figure 2). In the resulting face-on orientation, the π-π stacking direction is along the film normal while the chain direction is parallel to the rubbing direction [51] (Figure 2a). These studies showed not only that the anisotropy of μ_FET_ measured parallel and perpendicular to the rubbing direction depended on the intra-lamellar disorder [50], but also that the anisotropy of hole mobilities varied from 7 to 70, with the highest mobilities being measured along the rubbing direction, *i.e.*, along the long axis of polymer chain (and the highest anisotropies for the oriented face-on films) [51]. Control of polymer chain orientation with respect to the OFET substrate on large-scale was earlier established by treating the substrates with the silylating agent hexamethyldisilazane. This method was proven to promote phase segregation [52] and eventually self-organization [53] of P3HT chains into lamellar structure with two-dimensional conjugated sheets that were formed by inter-chain stacking. Depending on processing conditions, such lamellae could adopt edge-on and face-on orientations with respect to the substrate. Measured μ_FET_ mobilities were shown to differ by a factor of 100 and best-recorded μ_FET_ were obtained for the edge-on orientation and reached 0.1 cm^2^V^−1^s^−1^. These experiments highlighted the possibility of achieving high mobilities via two-dimensional transport (intra- and inter-chain) in self-organized conjugated lamellae, the mobility being limited by the π-π inter-chain rather than the intra-chain transport. Similar results of enhanced mobility were also reported when using self-assembled monolayers of octadecyltrichlorosilane (OTS) [54] covering the silicon oxide substrate on which the OFET is fabricated. In this case, μ_FET_ mobility of up to 0.02 cm^2^V^−1^s^−1^ was measured for a polyfluorene copolymer, representing an enhancement of 20-fold over the mobility on bare silicon oxide. It was suggested that the mobility enhancement mechanism was likely induced by the molecular interactions between the polymer and the OTS self-assembled monolayer.

Another interesting example of the highly anisotropic nature of charge transport in conjugated polymers is a study performed in strain-aligned P3HT [55]. For these systems, a charge mobility anisotropy of 9 was reached (the in-plane mobility was higher in the applied strain direction and decreased in the perpendicular direction), suggesting that the charge transport along the polymer chains within a crystal is strongly favored over the other crystallographic directions, including the inter-chain transport. This experimental observation was previously suggested in theory [56]. In addition, values of intra-chain mobility as high as ~0.1 cm^2^V^−1^s^−1^ were measured in that study. A slightly higher mobility μ_FET_ ~ 0.19 cm^2^V^−1^s^−1^ was reported around the same time by Chen *et al.* [2] when using aligned electrospun nanofibers of P3HT. The three orders of magnitude increase in mobility observed for fibers grown slowly compared to fibers grown in a rapid fashion at a higher shell flow rate was suggested to be due to the enhancement of the π-π stacking and the degree of crystallinity of P3HT chains in such nanofibers. Surprisingly, when tuning the degree of crystallinity of P3HT thin films via formation of aggregates in solution prior to deposition by application of low intensity ultrasound, a mobility of μ_FET_ ~ 0.03 cm^2^V^−1^s^−1^ was reported [21]. Although the authors provided strong evidence that the mobility depended on the degree of crystallinity of the P3HT, a relatively low mobility was found for all systems. This observation was ascribed to the complex multiphase morphology comprised of short, less ordered and long, better ordered nanofibrils that are embedded in an amorphous matrix. Such a picture would be consistent with more recent work where, also, ultrasonication was exploited to process P3HT. In this study, it was shown that the μ_FET_ of P3HT increased by one order of magnitude for material of higher molecular weight (from 1.16 × 10^−3^ cm^2^V^−1^s^−1^ to 2.73 × 10^−2^ cm^2^V^−1^s^−1^ for a molecular weight of 47.7 kDa), although no clear correlation between thin-film microstructure and macroscopic charge transport could be established [38].

Recently, Crossland *et al.* [6] have measured anisotropic in-plane mobility of P3HT films arising from a nanocrystalline spherulitic morphology in which charges were travelling either parallel to ordered π-stacked lamellar aggregates or perpendicular to them moving along individual chains before traversing amorphous inter-lamellar zones. A macroscopic μ_FET_ ~ 0.2 cm^2^V^−1^s^−1^ was measured for such architectures in the direction perpendicular to the ordered π-stacks, despite the periodic amorphous inter-lamellar interruptions. This was attributed to the fast intra-chain transport along tie-molecules spanning adjacent quasi-parallel lamellae. Similarly, high mobilities μ_FET_ of up to 6.2 cm^2^V^−1^s^−1^ were measured for self-assembled microribbons of liquid crystalline oligothiophene derivatives [57].

**Figure 3.**
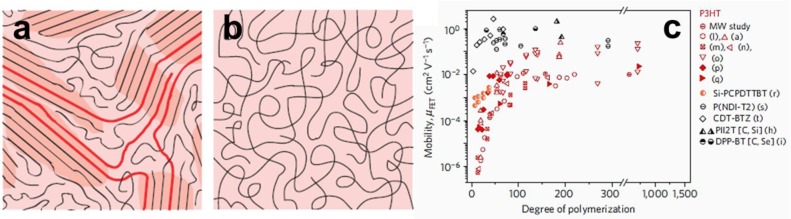
Schematics of the microstructure of (**a**) a semicrystalline polymer film and (**b**) a completely amorphous film as reproduced from Reference [67]. Note the coexistence of ordered (darker shadowed areas) and spaghetti-like amorphous regions. This microstructure is similar to the concept of fringed micelles. If the molecular weight is high enough and there is a large enough density of ordered material, long polymer chains (highlighted in red) can connect ordered regions without a significant loss of conjugation, greatly improving charge transport. (**c**) Mobility as a function of molecular weight for OFETs fabricated with P3HT (shown in red with different symbols referring to different studies) and a variety of other semiconducting polymers as reproduced with permission from Reference [67]. Copyright 2013, Macmillan Publishers Ltd: Nature Materials.

In contrast to the above reports, Lee *et al.* [58] have shown (using an anthradithiophene based molecule) the intra-spherulitic charge transport to be independent of the general direction of π-stacking. The fabricated OFETs exhibited comparable charge mobilities regardless of how their channels were oriented with respect to the general π-stacking direction [58]. Although the two studies used different materials (a polymer chain *vs.* a small molecule), the conclusions that can be drawn from them seem to indicate that the spherulitic morphology is already too complex when trying to clearly establish the charge transport properties. Single crystals of macromolecular semiconductors with unique molecular conformation and order (closely packed, π-π stacked, fully extended chains orthogonally oriented on the substrate) on all length-scales as schematically depicted in Figure 1 may, thus, be exploited in future to clearly establish the anisotropy of charge transport in conjugated polymers provided good crystal/electrode and crystal/gate dielectric interfaces can be created. Such model systems could be obtained via crystallization in solution by a self-seeding technique [59] and have already demonstrated remarkable absorption properties (a massive red shift of ~70 nm and a large energy separation of the vibronic peaks with respect to the reported vibrational spectra suggested a strong intra-chain interactions along the possibly fully delocalized chains due to the form-II packing) [60]. Moreover, performing conductive atomic force microscopy (C-AFM) in air on large single crystals made of a regioregular oligothiophene revealed a large anisotropy in the conduction with different charge mobility values that depended on the crystallographic structure of the single crystals [61]. The lower conduction was found to be in the direction of the π-π stacking (along the long axis of the single crystal) with a mobility value in the order of 10^−3^ cm^2^V^−1^s^−1^, and the higher one along the molecular axis (in the direction normal to the single crystal surface) with a mobility value in the order of 0.5 cm^2^V^−1^s^−1^. Note here that the mobility was extracted using the space charge limited current approach based on the current-voltage curves measured with the C-AFM. A solid platform for comparison exists. Relatively high OFET mobilities were measured for P3HT in crystalline nanoribbon networks [22], composited films [62], webs of conjugated nanofibers [63], supramolecular assemblies obtained by either adding poor solvent [64] or using block copolymers [3,65]. In these studies, μ_FET_ of up to ~0.08 cm^2^V^−1^s^−1^ were realized. Single crystals of poly(alkylthiophenes) (PATs) were also reported but μ_FET_ was still less than 10^−2^ cm^2^V^−1^s^−1^ [66]. Thus, a relation between microstructure of conjugated polymer films and resulting properties exists and it matters, for instance, if conjugated polymers films are semicrystalline or amorphous. Figure 3a,b schematically depict such examples of morphologies. It is also important to add in this context that the molecular weight of P3HT systems that are used in OFET fabrication can, in terms of mobility, be a limiting factor itself [67,68]. As can be seen from Figure 3c, higher OFET mobilities are in general measured for P3HT (and also other semiconducting polymers) for materials of higher molecular weight. It is worth noting here that all the above mobilities were measured mostly for neat polymer systems that were not blended, for example, with fullerenes. The influence of blending on charge mobilities is interesting as in blends, charges seem to become less mobile [69]. A comparison of the μ_FET_ mobilities measured for electrons and holes in neat P3HTand fullerenes as well as in P3HT/fullerene blends prepared using different solvents leads to interesting conclusions. Firstly, mobilities of both electrons and holes depend on the type of solvent that is used in preparations of thin films that are incorporated in the OFET devices (for example, *o*-xylene leads to higher electron mobilities than chlorobenzene when measured in neat fullerene films) [69]. Secondly, both electron and hole mobilities measured for pristine fullerene and P3HT respectively, are higher than the mobilities extracted for P3HT/fullerene blends [69]. This can negatively impact, for example, the overall efficiency of OPV devices as, faster charge carriers could migrate and be collected more efficiently at the collecting electrodes compared to the slower charge carriers that could recombine before reaching the electrodes.

Other conjugated polymers that have been widely employed for OFET fabrication are those based on p-phenylenevinylenes (PPVs) [5,70–72], including poly[2-methoxy-5-(2’-ethylhexyl)oxy-1, 4-phenylenevinylene] (MEH-PPV). While their charge-carrier mobilities are relatively modest (the highest mobility so far has been μ_FET_ = 0.8 × 10^−2^ cm^2^V^−1^s^−1^, measured for free standing, ordered PPV sheets), certain structure/property interrelationships can be deduced. Anisotropy considerations apply, for instance, to planar ladder-type PPVs, where the intra-chain mobility measured by microwave conductivity was μ_MC_ = 30 cm^2^V^−1^s^−1^ in solid samples [73]—a value that is four orders of magnitude higher than μ_FET_ as the latter is limited by inter-chain charge hopping.

Over the last few years, a large library of new materials have been synthesized, often based on donor-acceptor copolymers, many with charge-carrier mobilities exceeding 1 cm^2^V^−1^s^−1^; for instance, a mobility μ_FET_ = 5.5 cm^2^V^−1^s^−1^ was obtained for single conjugated fibers [4]. The structural features of these polymer layers seem to be divers and a high charge-carrier mobility is attributed to structures with an enhanced π-π stacking [20], increased intermolecular interactions [19], (edge-on) lamellar arrangements [74,75], high film crystallinity [76,77] and/or molecular orientation/order [1,76,78]. While many open questions, thus, still remain, it is clear from the above that experimental data point towards an anisotropic nature of charge transport in macromolecular semiconductors. For more details regarding a general relationship between lattice order/disorder, aggregation/molecular packing and charge transport from both theoretical and experimental point of view, the reader is encouraged to consult the excellent work by Nan *et al.* [35] and Noriega *et al.* [67]. In the latter work, a unified model is proposed of how charge carriers travel in conjugated polymer films showing that in long, conjugated chains short-range inter-molecular aggregation is sufficient for efficient long-range charge transport [67]. The limiting charge transport step is thereby trapping caused by lattice disorder.

## 3. Influence of Molecular Conformations and Long-Range Arrangement on the Absorption and Photoluminescence Properties of Active Layers of Conjugated Polymers

Absorption of a photon by a conjugated polymer generates a bound electron-hole pair called an exciton with a binding energy typically around hundreds of meV [79]. Oppositely, the decay of, for example, an exciton in a material possessing emitting properties leads to the emission of a photon. This latter mechanism is exploited in OLEDs; it significantly depends on the molecular conformation adopted by the conjugated chains [80,81] just like their absorption behavior does [81], hence, processing is of paramount importance to obtain the desired functionality.

MEH-PPV has for a long time been one of the most widely studied polymers for OLED applications. Early experimental evidence of how the performance of MEH-PPV-based OLEDs depends on thin-film microstructure was produced by Nguyen *et al.* [82]. These authors demonstrated that intra-chain species exist in thin films of conjugated polymers and that the nature of intra-chain interactions can be controlled by selection of processing conditions and, thus, the thin-film molecular arrangement of the active species. In later studies, it was shown that MEH-PPV chains can adopt a more ordered, rather well-packed arrangement characterized by a longer conjugation length (determined by the number of coplanar conjugated rings) leading to a spectral red shift in both absorption and emission [83]. The idea that molecular features matter for the optical response of conjugated polymers was supported by other studies. For example, comparing PPV-based polymers that were amorphous and adopted layered structures, Rathgeber *et al.* [84] have shown, in 2010, that the emission became more blue shifted when the π-π stacking was increased. No obvious correlation of the emission peak position and the degree of order of the π-π stacking was however found.

**Figure 4.**
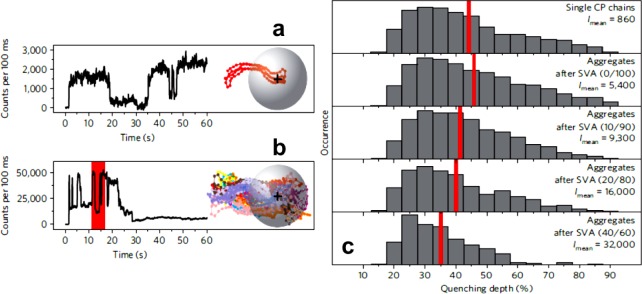
Representative single-particle fluorescence transients and quenching depth histograms as adapted from Reference [45]. (**a**) A single MEH-PPV chain and (**b**) an MEH-PPV aggregate in a poly(methyl methacrylate) host matrix are shown under the same experimental conditions. The inset is a schematic representation of a single conjugated polymer chain and an aggregate consisting of 25 conjugated polymer chains with a fluorescence quencher; (**c**) histograms of fluorescent quenching depths of blinking for different single MEH-PPV particles in an ensemble. The red line represents the mean quenching depth. The processing condition and the mean intensity *I_mean_* of the particles is denoted in the upper right corner. Reprinted with permission from Reference [45]. Copyright 2011, Macmillan Publishers Ltd: Nature Materials.

Fully correlating chain conformation, order and packing with the emission properties of conjugated polymers and manipulating them in a controlled way is a challenge that has remained to this date. This is especially a demanding task for macromolecular semiconductors where the molecular arrangement from the nano- to the micron-range is very sensitive to the preparation/processing methods selected. An illustrative example of this is the observations made when exposing MEH-PPV to solvent vapor. The polymer chains can in such a scenario easily change their conformation and packing via folding and unfolding from, e.g., a coil-like conformation to highly ordered, more extended conformations [85]. MEH-PPV aggregates with volumes of at least 45.000 nm^3^ embedded in a poly(methyl methacrylate) host matrix and comprising chains of highly ordered conformations have been found to display pronounced fluorescence blinking behavior (*i.e.*, switching between bright and dark states of the emitting MEH-PPV when continuously photoexcited) as revealed by single-molecule spectroscopy [45]. This was attributed to a long-range energy transport [45] that can extend up to 75 nm in such ordered aggregates [28]; *i.e.*, blinking is occurring because the excitation of an individual chain is localized, hence only one or a few conjugated segments can reemit this energy. Blinking and typical single-particle fluorescence transients acquired using time-resolved wide-field fluorescence microscopy can be seen in Figure 4a,b. Fluorescence quenching seems to depend on the number of MEH-PPV chains forming an aggregate as revealed by the quenching depth measurements shown in Figure 4c (quenching depth decreases slightly with increasing size of the MEH-PPV aggregates). Moreover, it was shown using low-molecular weight PPV derivatives that the conformation of an individual segment or chromophore (that is a distinct conjugated region on the polymer chain characterized by delocalization of π-electrons [86]), and not necessarily that of the entire polymer chain, controls the resulting photophysical properties [87].

Theoretical studies based on molecular dynamics were also performed to explore the relation between the structure/molecular packing and optoelectronic properties of MEH-PPV, including localization and delocalization [88,89], *i.e.*, the distance over which π-electrons can be shared. The electronic structure was found to depend on the conformational order of the individual chains with little effect of inter-chain coupling, indicating different energy transport mechanisms along and across polymer chain [89]. Conversely, long-range inter-molecular interactions and sparse packing were ascribed to be responsible for the formation of multiple, highly localized trap states in amorphous MEH-PPV [88].

**Figure 5.**
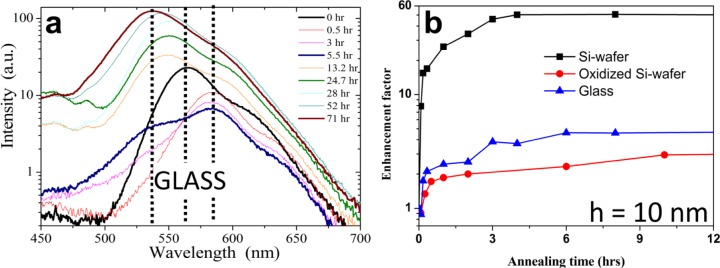
(**a**) Temporal evolution of the PL emission of a 20 nm thick MEH-PPV/PS film during dewetting on glass; (**b**) the temporal evolution of the PL-enhancement factor for films of MEH-PPV/PS (of thickness h = 10 nm) in the course of annealing and dewetting for films prepared on different substrates. Reprinted with permission from Reference [91]. Copyright 2013, American Chemical Society.

Beneficially, inter-chain interactions can be significantly suppressed by “diluting” conjugated polymer chains in an optically inert matrix of, for example, polystyrene (PS), providing a tool to manipulate exciton delocalization/localization. To give an illustrative example: a significant spectral blue-shift is observed upon dilution of MEH-PPV in PS. Intriguingly, the PL efficiency can be doubled for certain MEH-PPV/PS weight ratios and, when integrated in OLEDs, the devices display drastically higher electroluminescence efficiencies compared to neat MEH-PPV films [90]. Furthermore, this enhancement in PL of such blends can be significantly enhanced using a dewetting process whereby the MEH-PPV chains are extended via strong shearing forces [91]. The PL emission can be further manipulated via the annealing of MEH-PPV/PS thin films because this procedure affects the molecular conformation of the conjugated component (see Figure 5a). Prior to dewetting, annealing leads to a significant decrease of PL intensity combined with a red-shift, indicating chain aggregation and likely an increase in conjugation length because of this treatment. When dewetting sets in, however, a new, blue-shifted PL emission peak appears. Its intensity increases by 50 times, compared to the non-annealed samples, when a silicon substrate is used (Figure 5b). This effect is not observed when other substrates, such as oxidized silicon or glass are employed, indicating the relevance of the film/substrate interface in this phenomenon.

An interesting example how the molecular conformation is affected by certain optoelectronic processes is given by observations made on paraphenylene oligomers upon photoexcitation; while the ground-state is non-planar, a planar structure is formed in the excited state [92]. This planarization is reported to be accompanied by a slight red-shift [92] but also macroscopically detectable changes in material properties such as viscosity [93] due to a collective behavior of a number of molecules.

Structural changes can also lead to other effects—one is the so-called polychroism phenomenon. Here, depending on processing parameters, PL can be emitted from a high-energy state (the so-called blue phase) or from a low energy state (the so called red phase) [94]. Measuring the absorption and PL in solution as a function of parameters like temperature and concentration, a transition from blue to red phase with the corresponding changes in conjugation lengths can be observed [94].

Similar phenomena can be exploited in polymers used in OLED applications, such as polyfluorene (PFO). Khan *et al.* [42] have, for example, shown through control of the fraction of planarized β-phase within the disordered glassy PFO film that the chains adopting a β-phase conformation feature a higher delocalization of excited states compared to some of the other conformations PFO chains can adopt. As a consequence, a significant fraction of the PL emission is originating from these few percent of planarized chains embedded in the disordered glassy phase [42]. The absorption, in contrast, was found to be dominated by the majority phase, *i.e.*, the amorphous matrix. In agreement with these findings is the fact that the PL emission was found to red-shift and broaden when changing the molecular conformation of the PFO chains by increasing the pressure during the measurement [18]. This red-shift in PL emission was therefore attributed to a reduced inter-chain distance [95]. This is in agreement with the observation that molecularly more oriented architectures such as PFO nanofibers obtained by electrospinning showed highly polarized PL emissive properties. These maybe exploited in applications like OLEDs [17] or security features. Similar structure/optoelectronic property relationships in terms of absorption and PL were also probed using P3HT [96–99], water soluble P3HT [100], other thiophene based block copolymers [101], co-oligomers [102], or even systems like donor-acceptor semicrystalline polymers [103], and alternating co-polymers with fluorine and thiophene moieties [104]. For example, by means of temperature-dependent absorption and PL spectroscopy, it was shown that the optical emission is dominated in regioregular P3HT by weakly coupled H-aggregates (characterized by the inter-chain coupling of polymer chains) while the relative absorbance of the 0–0 and 0–1 vibronic peaks can be used to extract the intermolecular coupling energy in thin P3HT films cast from various solvents [96]. Similarly, employing wavelength and time-resolved PL studies of isolated P3HT nanofibers cast on glass substrate from suspensions of less and more regioregular P3HT, multiple vibronic replicas that appeared to be associated with the existence of both H- and J-type aggregates (characterized by the intra-chain coupling along a single polymer chain) were observed. PL spectral measurements on nanofibers made from more regioregular P3HT showed a red-shifted electronic origin, an increased 0−0/0−1 PL intensity ratio for the J-type aggregates, suggesting an enhanced structural coherence length and intra-chain order [97]. Moreover, absorption, fluorescence emission, and Raman spectroscopy of dilute nanofibers in toluene dispersions revealed that P3HT chains exhibit long-range intra-chain order that suppresses the inter-chain exciton coupling and that a delicate interplay exists between intra-chain order and inter-chain coupling as concluded from comparing the emission 0−0/0−1 vibronic intensity ratios [98]. However, in most of these cases, applications of, for example, P3HT in OLEDs are rather limited. Nonetheless, it is worth noting that studies of absorption and emission of P3HT can be very effective methods to explore the charge separation process (*i.e.*, how efficient the exciton dissociation is) when mixing P3HT with fullerenes and creating blends that are used in OPV device fabrication. The absorption spectra in thin films made of such blends suffer significant changes, *i.e.*, a decrease/quenching of the inter-band absorption in the wavelength range of 450–600 nm is usually observed for this particular system [105]. This absorption quenching was attributed to both the disordering of the P3HT chains when adding fullerenes and a charge transfer between the P3HT and fullerenes. PL properties also change in such blends and PL quenching is generally strong evidence for charge transfer [47], *i.e.*, the most significant the PL quenching is, the most efficient the charge transfer is.

In summary, different types of defects that can occur in organic semiconductors, including conformational torsional angle misfit or various chain kinks lead to localization of π-electrons, as has been convincingly shown on paraphenylene oligomers [106]. Thereby, localization of π-electrons over a multitude of distances due to defects is translated into a multitude of absorption and PL responses [107]. More information on the methods used to control structure and morphology from the molecular to the macroscopic level and how chain conformation influences the resulting optoelectronic and photophysical properties of active layers of conjugated polymers can be found in the literature in several well documented reviews [108–111]. For a comparison of the impact of molecular conformation on the optical properties between the experimental observation and theory the reader is referred to [112].

## 4. Structural Influences on the Photovoltaic Properties of Polymer: Fullerene Bulk-Heterojunctions

Since the first bulk heterojunction (a mixed donor-acceptor active layer that relies on random phase separation of the constituents and usually exhibits significant structural disorder; BHJ), based on MEH-PPV, was reported [9], the development has been rapid and device efficiencies reach now up to 10% [113–115] with fundamental theoretical descriptions suggesting an upper limit of 20%–24% [114]. Factors limiting an efficient conversion of photons to electricity in organic (and hybrid) materials are related to light absorption [116,117] and other internal phenomena like exciton dissociation [118,119], various complex photophysical processes [120], as well as charge-carrier transport and collection [117,121]. Improved electrode design, use of interlayers and/or more complex device geometries have also led to this fast evolution, however, for reasons of space, we will not focus here on these latter considerations.

BHJ have been proposed as an alternative to planar structures to provide architectures that can efficiently dissociate photogenerated excitons [118]. Various concepts have thereby been developed, many being based on blending a semiconducting polymer with either fullerene derivatives [122,123] (this is the type of solar cells we will discuss here), other polymers [124–126], nanocrystals [127–129], or combining it with inorganics to form a hybrid solar cell [130]. Independent of the approach selected, it has become rapidly evident that donor-acceptor BHJ devices rely on various internal processes. Thereby, disorder on all length scales can result in losses, e.g., due to exciton recombination, poor charge extraction, *etc.* In order to develop high-performance OPVs, active layers of controlled phase morphology and microstructure (and possibly molecular conformations) on the nano- to microscale are needed [131–134].

**Figure 6.**
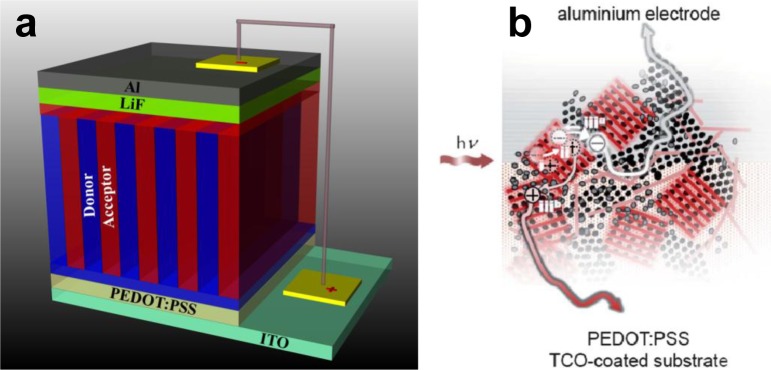
(**a**) Schematic 3D representation of an OPV based on an architecture that has been proposed to be ideal for exciton dissociation and charge collection; (**b**) illustration of the function model proposed by Jamieson *et al.* [142], where excitons are generated and separated in a finely mixed polymer/fullerene phase, while spatial separation of the electron and holes necessitates localization of the electrons in the aggregated fullerene domains. Thereby, the energy offset between the lower unoccupied molecular orbital (LUMO) levels of the fullerene in the mixed and relatively phase-pure domains provide an energy difference localizing electrons in the fullerene regions (reprinted with permission from Reference [142]). Copyright 2011, Royal Society of Chemistry.

Due to the rather short exciton diffusion distances in organic materials (around 10 nm [79,135]), it has been proposed that exciton generation has to occur within ~10 nm of a donor–acceptor (D–A) interface; this includes molecular interfaces between molecules (e.g., in an intermixed phase or co-crystal) or, on longer length scales, between domain boundaries of phase-pure domains of the two components. At such interfaces, the exciton can separate into a positive and a negative charge that subsequent should be extracted via continuous and direct pathways to the respective electrodes [134,136,137]. Due to this requirement of close proximity of the donor/acceptor interfaces, an architecture, as depicted in Figure 6a, has been suggested to be an optimum BHJ structure [47,138,139]. It consists of 10 nm wide, alternating domains of, donors and acceptors, orthogonally oriented with respect to the substrate. A considerable amount of effort had been devoted to the creation of such an idealized morphology, e.g., through use of controlled crystallization processes [47,140]. Recently, however, evidence was collected that intermixed phases are formed by the donor and acceptor. These domains were suggested to play an important role in charge dissociation—provided that relatively pure, molecularly ordered phases of at least one of the components were present. Indeed, in specific systems, aggregated fullerene domains have been postulated to be the key factor driving the spatial separation of photogenerated electrons and holes from these intermixed regions because fullerene aggregates exhibit an increased electron affinity, providing an energetic driving force for spatial separation of electrons and holes. Thus, it seems increasingly clear that a functional model, based upon charge generation in a finely intermixed polymer/fullerene phase, followed by spatial separation of electrons and holes at the interface of this mixed phase with crystalline fullerene domains (Figure 6b), might be relevant for efficient photovoltaic performance of organic donor/acceptor systems [141,142].

In addition to the phase morphology (*i.e.*, the number of phases, their nature, and distribution) of BHJ films, other structural factors have been reported to affect their performance. For P3HT/fullerene BHJs, possibly the most widely studied systems, Kim *et al.* [122] showed that the regioregularity of the donor polymer is critical for the performance of the resulting devices due to its influence on the molecular organization of P3HT chains that leads to better ordered architectures of improved transport properties. A range of other parameters related to the phase behavior and structural features of OPV blends have become evident to play an important role in device performance, including the miscibility of the two components and their aggregation behavior [140,143,144], their structural organization and self-assembly/crystallization [11,47,140,145,146], their degree of crystallinity [143,147], grain boundaries present in the blend films [147], type of materials and their intermolecular packing [148], the nature of the D-A interface [149], as they can significantly influence various internal processes that take place in the active layers, such as exciton diffusion, charge separation/dissociation, charge carrier recombination, and migration. Below, we will discuss a few of those features.

Crystallization of at least one component often enhances the performance of OPVs [145,146] unless the crystalline domains become too large (limiting the exciton dissociation and eventually favoring the charge recombination). Then, the opposite effect can occur. Indeed, increasing the crystalline content of the polymer in P3HT/fullerene blend increases the device efficiency [150] as long as the P3HT domains are interconnected enabling charge transport. Complete exclusion of the fullerene will, however, reduce the charge generation efficiency and therefore, the device efficiency [150]. That is why, during processing of active layers, one has to consider a range of processes including the evaporation of residual solvent, the relaxation of molecular conformation previously adopted by the conjugated chains, crystallization of the components in the blend, *etc.* [151]. Additional processing parameters can further influence the type of molecular structures forming in the BHJ and their final size. For example, thermal annealing of the BHJ [152], the type of solvent [153], as well as the use of a processing additives [154], were shown to be of critical importance for efficient OPVs as all can influence the crystallization process (the tendency of P3HT chains to crystallize is generally disturbed by the addition of fullerenes), *i.e.*, the resulting polymer chain arrangement within the BHJ, and, therefore, the overall device efficiency.

A good illustration of this interplay of a range of processes in OPV blends comprising P3HT nanofibrils and a fullerene derivative is obtained when long fibrils of almost 100% crystallinity are used. The resulting blend structures showed 6.5 times higher in-plane conductivity [11]. Incorporation of such active layers in OPV devices using a roll-printing method resulted in comparable device performance to that of blends comprising a usual P3HT component. What is interesting is that the OPV efficiency of the devices made of P3HT nanofibrils did not decrease when the active area was enlarged. Clearly, various device features depend on the BHJ structure and many have a complex interdependence that will not be discussed here. Further valuable information on the control of BHJ OPVs based on blends can be found in the literature [155,156].

When an exciton reaches the D-A interface, so-called charge transfer (CT) states form. These can recombine either via geminate recombination or with charges located on adjacent molecules (via bimolecular recombination). These processes are undesirable for OPV performance. Indeed, for OPVs it is important that such CT states dissociate and separate via charge separated states in order to generate charge carriers (electrons and holes) that can migrate towards the electrodes producing electricity. The nature of such CT states—hot or cold—and the role they play in OPV performance are currently strongly debated. What is clear is that all these phenomena of recombination and/or charge transfer are highly dependent on the morphology and molecular arrangements at the D-A interface [157]. It seems for example, that better ordered BHJs exhibit lower rate of geminate recombination, that is, more charge carriers are obtained (implicitly, a higher efficiency of resulting OPVs can be expected). More detailed information on such rather complex processes can be found in the literature [27,158].

**Figure 7.**
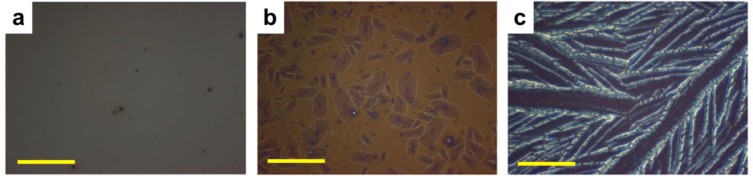
Optical microscopy images of a PCDTBT:PC_71_BM (1:4) film on a silicon substrate that are annealed for one hour (**a**) 130 °C; (**b**) 155 °C and (**c**) 200 °C. The scale bar in all images represents 20 μm. This figure was adapted with permission from Reference [162]. Copyright 2012, John Wiley and Sons.

Regarding the progress made on the materials development side, a large library of novel conjugated OPV polymers, mainly “push-pull” copolymers, have recently been reported. Devices based on such copolymers blended with fullerenes led at first to certified power conversion efficiencies of up to 6.88% [159]. The improvement in device efficiencies was, for certain systems, attributed to a favorable microstructure. Using alternating co-polymers based on thieno[3,4–*b*]thiophene (TT) and benzodithiophene (BDT) repeat units, for instance, active layers comprised of hierarchical architectures comprised of crystallites of several nanometers, aggregates of tens of nanometers, and polymer and fullerene domains of hundreds of nanometers that co-exist, were reported [160]. The resulting OPVs showed an efficiency of 7.4%. Recently, Zhou *et al.* [12] have shown that the performance of such OPVs can be further enhanced up to 7.9% efficiency by using a solvent treatment consisting of spin casting methanol solvent on top of the active layers. This performance was shown to originate from an increase in built-in voltage across the device due to passivation of surface traps and a correspondingly increase of surface charge density [12]. Promisingly, using an alternating co-polymer, such as poly[*N*-900-hepta-decanyl-2,7-carbazole-alt-5,5-(40,70-di-2-thienyl-20,10,30-benzothiadia-zole)] (PCDTBT), blended with fullerenes, BHJ OPVs with internal quantum efficiency approaching 100% were reported, which would imply that almost all absorbed photons resulted in separated pairs of charge carriers that are then all collected at the electrodes. The corresponding power conversion efficiency is 6.1% [161]. Note, though, as for the “older” systems, such as P3HT/fullerene blends, selection of solvent or substrate was found to be important. For the PCDTBT system this was attributed to the fact that its glass transition temperature is affected by, e.g., solvents; as a consequence, the π-π stacking of the polymer can be disrupted depending on the choice of processing conditions [162]. For example, annealing thin films of PCDTBT:PC_71_BM ([6,6]-phenyl-C_71_-butyric acid methyl ester) blend at different temperatures induces different structural ordering of molecules (Figure 7) via coarse phase separation/crystallization that are visible under the optical microscope; in contrast, at lower annealing temperatures, the induced molecular structures are not visible at the micrometer scale which seems to be beneficial for the morphological stability of this type of blends used in OPV devices [162].

Other interesting blocks that have been explored in such “push-pull” polymers include thieno[3,4–*c*]pyrrole-4,6-dione (TPD)- and 1,3-bis(thiophene-2-yl)-5,7-bis(2-ethylhexyl)benzo-[1,2–*c*’] dithiophene-4,8-dione (BDD)-moietites [144,147,163,164]. When used in combination with fullerenes, such copolymer, coarsely phase separated upon solvent annealing is leading to OPVs with an efficiency of 4.99% [163]. Optimizing the initial solution resulted in the formation of smaller domain sizes and an increased device efficiency (6.67%) [144]. Adding additives to this polymer/fullerene blend was, furthermore, reported to improve the degree of crystallinity, further increasing their efficiency to 7.3% [147].

More recently, poly(benzo[1,2−*b*:4,5−*b*′]dithiophene–thieno[3,4−*c*]pyrrole-4,6-dione) (PBDTTPD) polymers with branched side chains have been synthesized with the objective to have further means to manipulate the self-assembly process in blends with the fullerene. Indeed, OPVs of a power conversion efficiency of 8.5% have been reported with this system [164].

Summarizing this part, it is clear from the above brief overview that many structural features play a significant role in organic BHJ. Establishing valid structure/processing/property interrelationships is rendered even more complex due to the multicomponent nature of OPV active layers. Hence, many important issues related to the various internal processes (including the charge separation at the donor-acceptor interface) have not been discussed here. We direct readers to further comprehensive work that can be found in the literature [165–170]. Here, the reader can further understand both from theoretical and experimental point of view what the role of molecular architecture is and how the intermolecular interaction between the donor and acceptor components is influencing the charge separation and charge transport processes taking place in the OPVs. Nanoscale morphologies exhibiting high D-A interface area, comprised of D and A domains matching the size of exciton diffusion length and possessing percolating elements throughout the whole BHJ that can be used as “fast lanes” for efficient charge transport will positively impact the internal processes from exciton diffusion, to exciton dissociation and charge separation, to carrier migration, finally, leading to OPV devices of better overall efficiency. Additionally, this literature reveals the main challenges and the various limitations of OPV devices.

## 5. Conclusions

There is a large variety of processing aspects, including selection of solution preparation methods, nature of solvent or substrates used for solution or thin film preparation, choice of film deposition method and post-deposition treatment procedures such as annealing (e.g., solvent annealing *vs.* heat treatment), use of methodologies that lead to molecular ordering (including directed or high-pressure crystallization), *etc.* that can be exploited to modify the molecular structure and (local) morphology in the active layers of organic devices, and therefore can improve or alter the resulting optoelectronic properties of such structures. As a result, the device performance is strongly intercorrelated with the processing parameters and environment selected. This diversity of processing options and tools, combined with a lack of model systems (for instance, systems characterized by fully extended conjugated polymer chains with unique molecular orientation/conformation of all constituent molecules on multiple length scales) leads to the difficulty to unambiguously establish relevant structure/optoelectronic property relationships; including the question at what conditions intra- or inter-chain transport dominates. Clearly, this diversity of processing parameters is responsible for the various and sometimes contradicting results reported in literature but it is also the key to gain in-depth insight in relevant interrelationships that can assist in improving the optoelectronic properties of macromolecular semiconductors further. For this methods need to be developed to *routinely* create macroscopic model systems of *fully controlled molecular order* on multiple length scales that can be further used to evaluate more precisely the structure/optoelectronic properties relationship.
